# Heterochrony and Oophagy Underlie the Evolution of Giant Filter‐Feeding Lamniform Sharks

**DOI:** 10.1111/ede.12496

**Published:** 2024-11-30

**Authors:** Joel H. Gayford, Duncan J. Irschick, Andrew Chin, Jodie L. Rummer

**Affiliations:** ^1^ College of Science and Engineering James Cook University Townsville Queensland Australia; ^2^ Shark Measurements London UK; ^3^ Department of Life Sciences Imperial College London London United Kingdom; ^4^ Department of Biology, Morrill Science Center University of Massachusetts Amherst Massachusetts USA

**Keywords:** body size, development, *Elasmobranchii*, gigantism, paedomorphosis

## Abstract

Evolutionary transitions toward gigantic body sizes have profound consequences for the structure and dynamics of ecological networks. Among elasmobranchs (sharks and rays), gigantism has evolved on several occasions, most notably in the iconic Megalodon (*Otodus megalodon*†) and the extant whale shark (*Rhincodon typus*), basking shark (*Cetorhinus maximus*), and megamouth shark (*Megachasma pelagios*), all of which reach total lengths exceeding 6 m and, in some cases, reach 21 m or more. Comparative phylogenetic studies suggest that filter feeding and heterothermy provide two alternative evolutionary pathways leading to gigantism in sharks. These selection‐based explanations for gigantism are important; however, our understanding of evolutionary transitions in body size is fundamentally constrained without a proximate, mechanistic understanding of how the suite of adaptations necessary to facilitate gigantism evolved. Here we propose the heterochrony hypothesis for the evolution of the giant filter‐feeding shark ecomorphotype. We suggest that craniofacial adaptations for oophagy in embryonic stages of lamniform sharks are retained through ontogeny in *C. maximus* and *M. pelagios* by paedomorphosis, resulting in an enlarged head and mouth relative to the rest of the body, even in adulthood. This change in developmental timing enables these taxa to optimize prey acquisition, which is thought to be the limiting factor for the evolution of gigantism in filter‐feeding marine vertebrates. We discuss the concordance of this hypothesis with current developmental, morphological, and evolutionary data, and we suggest future means by which the hypothesis could be tested.

## Graphical abstract

Retention of juvenile craniofacial characteristics by paedomorphosis (heterochrony) may have facilitated the evolution of gigantism in filter‐feeding lamniform sharks, by increasing the efficiency of prey acquisition.

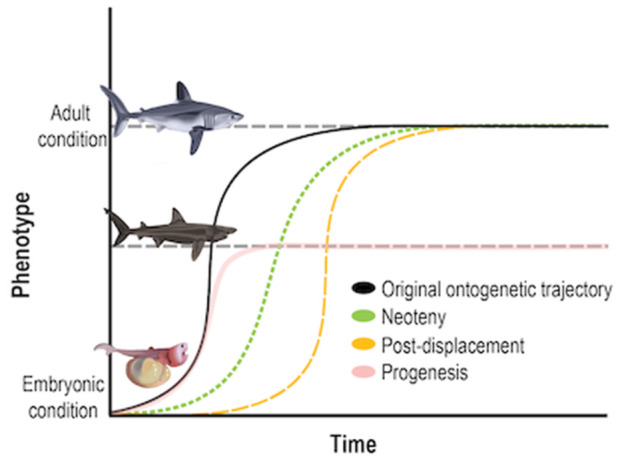

## 1 Introduction

Elasmobranchs (i.e., sharks and rays) have been an ecologically important component of marine biodiversity since the clade's origin (Heithaus et al. 2010; Maisey, Naylor, and Ward 2004; Maisey 2012). The range of ecological niches filled by elasmobranch taxa is in part due to an impressive degree of variation in body size, with extant species varying between ~20 cm and ~21 m in total length (Ebert, Dando, and Fowler 2021). Gigantic body sizes (> 6 m maximum total length) have evolved independently on several occasions in elasmobranch lineages that differ radically in ecological lifestyle (Ferrón, Martínez‐Pérez, and Botella 2018; Pimiento et al. 2019). The presence of gigantic elasmobranchs in marine ecosystems has important consequences for energy fluxes and the stability of trophic networks (Ferrón, Martínez‐Pérez, and Botella 2018). Prominent examples include apparently heterothermic macropredators, such as the white shark (*Carcharodon carcharias*) and the extinct ‘Megalodon’ (*Otodus megalodon*†), as well as filter‐feeders, such as the giant oceanic manta (*Manta birostris*), megamouth shark (*Megachasma pelagios*), basking shark (*Cetorhinus maximus*), and whale shark (*Rhinocodon typus*). These taxa are symptomatic of a broader dichotomy in gigantic marine vertebrates between species with relatively low metabolic demands that consume vast quantities of smaller prey, and highly active macropredators hunting small numbers of large, energy‐rich prey (Goldbogen and Madsen 2018; Pimiento et al. 2019; Vermeij 2016). Consequently, it is thought that gigantism in elasmobranchs, cetaceans, and other marine vertebrates is facilitated by diverse adaptations for maximizing energy intake (Ferrón, Martínez‐Pérez, and Botella 2018; Goldbogen and Madsen 2018; Pimiento et al. 2019).

Several recent studies have addressed the evolution of body size in cartilaginous fishes (Marion, Condamine, and Guinot 2024; Mull et al. 2024; Pimiento et al. 2019), focussing on ultimate, adaptive explanations that frame evolutionary transitions in body size in terms of natural selection and constraint. Whilst these studies are certainly valuable, they are not the only lens through which to view the evolution of gigantism. Proximate explanations, addressing the mechanistic and/or developmental basis of gigantism in sharks have not received the same attention. This is undoubtedly due to the logistical challenges associated with developmental studies in gigantic taxa, and the scarcity of ontogenetically complete fossil records. However, to truly understand the evolution of gigantism in sharks (as in any other taxon), it is necessary to integrate both proximate and ultimate explanations (Thierry 2005). Increasingly, evolutionary–developmental (evo–devo) approaches have been applied to study the proximate basis of morphological variation, including the nature of the underlying gene regulatory networks (Alberch 1980; Carroll 2008; Irschick et al. 2013; Mallarino and Abzhanov 2012). Among the insights provided by this evo–devo approach, it is clear that much variation in organismal shape and size across vertebrate and invertebrate diversity is driven at least in part to changes in developmental timing, a phenomenon known as heterochrony (Dobreva, Camacho, and Abzhanov 2022; Koyabu et al. 2014; McNamara 2012; Werneburg and Sánchez‐Villagra 2015). What, if any, role heterochrony might play in the evolution of body size disparity among sharks has yet to be addressed.

Here, we briefly review what is known about the evolution of gigantism in elasmobranchs, focussing on the evolution of the gigantic filter‐feeding ecomorphotype. Subsequently, we propose the heterochrony hypothesis for the evolution of giant filter‐feeding sharks. We suggest that paedomorphosis (in which juvenile characteristics are retained into maturity) in oophagous lamniform sharks may have resulted in the evolution of unique morphological specializations for filter feeding, as seen in *M. pelagios* and *C. maximus*, that directly facilitated gigantism in these taxa. We consider the extent to which this hypothesis is supported by existing developmental and ecological data and provide potential mechanisms through which it could be tested in future studies.

## 2 The Evolution of Giant Filter‐Feeding Sharks

Filter feeding – the capture and consumption of small prey items suspended in the water column – has evolved on numerous occasions in diverse vertebrate and invertebrate marine lineages, and it is generally associated with increased size relative to non‐filter‐feeding ancestral taxa (Stiefel 2021). In sharks, filter‐feeding evolved independently on at least four occasions – once in each of Aquilolamnidae†, Megachasmidae, Cetorhinidae, and Rhincodontidae (Friedman et al. 2010; Misty Paig‐Tran and Summers 2014; Pimiento et al. 2019). Additional putative filter‐feeding taxa are known from fossil remains (e.g., *Pseudomegachasma*†, Shimada et al. 2015); however, phylogenetic uncertainty precludes us from determining whether such evidence represents an additional independent evolution of filter‐feeding. Fossil records and paleoecological data indicate that turnover and diversification trends of other lineages – namely, the increase in planktonic primary productivity and the extirpation of both gigantic filter‐feeding actinopterygians and macropredators – created the ecological conditions necessary for the evolution of filter‐feeding gigantism in sharks (Friedman et al. 2010; Pimiento et al. 2019).

**Figure 1:**
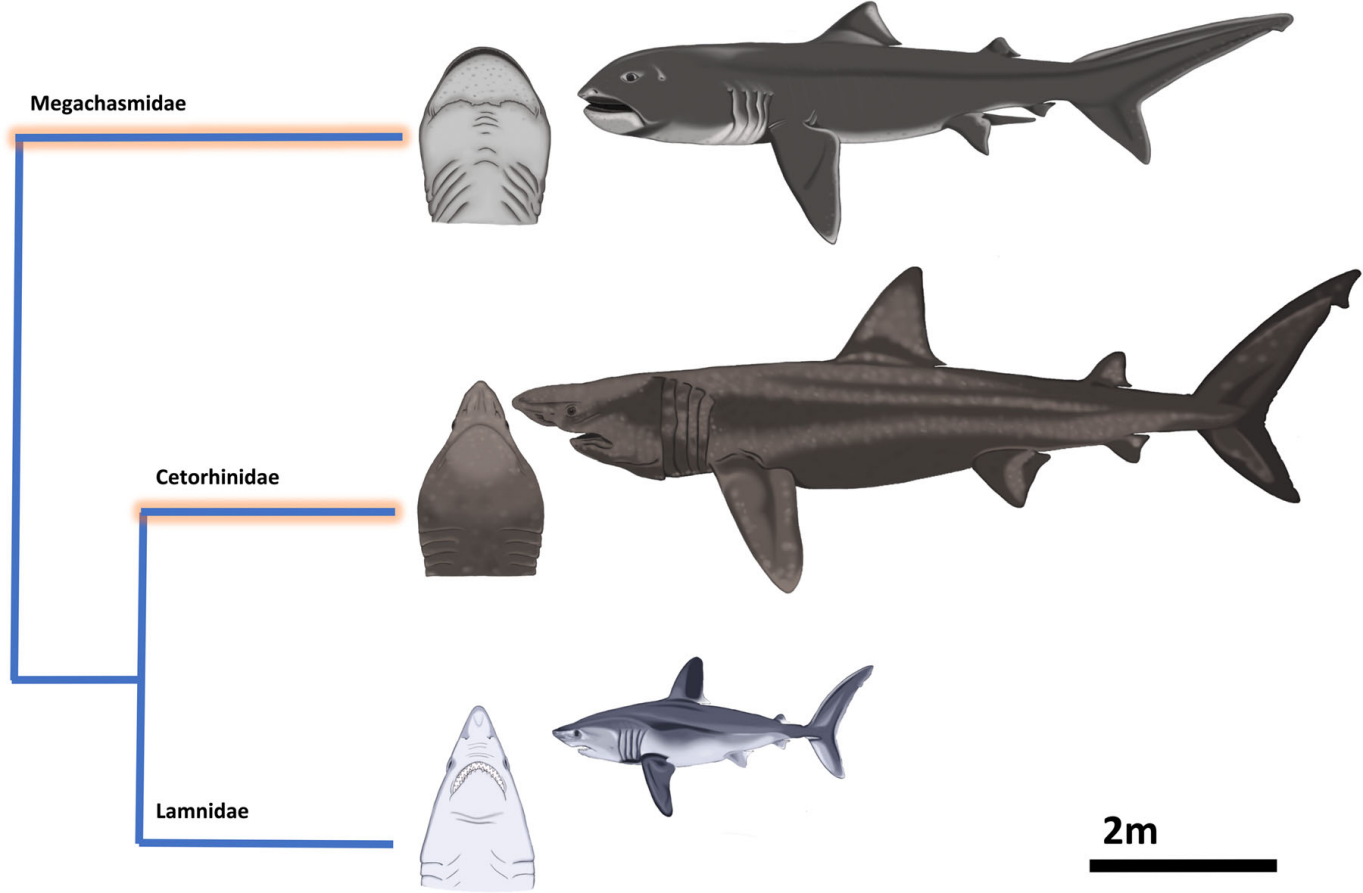
*Megachasma pelagios*, *Cetorhinus maximimus*, and *Lamna nasus* (a ‘typical’ lamniform shark, included here for comparative purposes) in lateral view, and the head of each species in ventral view, highlighting the extreme craniofacial adaptations of filter‐feeding taxa. Full body illustrations are drawn to scale with one another, however, head illustrations are not drawn to scale, the cladogram is not time‐scaled, and branch lengths are arbitrary. [Color figure can be viewed at wileyonlinelibrary.com]

The sequence of trait evolution leading to both gigantism and filter‐feeding in Megachasmidae, Cetorhinidae, and Rhincodontidae remains uncertain, as does the potential interplay between filter‐feeding and other drivers of gigantism, such as heterothermy (Pimiento et al. 2019). *C. maximus*, for example, exhibits both filter feeding and heterothemy (Dolton et al. 2023), further complicating our understanding of the role each trait has played in shark body size evolution. It is important to recognize that the ‘filter‐feeding habit’ of these taxa represents a suite of behavioral and morphological adaptations to maximize performance and prey acquisition (Cade et al. 2020; Yun and Watanabe 2023; Wilga, Motta, and Sanford 2007). Amongst the three extant filter‐feeding giants, different combinations of traits appear to facilitate successful filter feeding: both *C. maximus* and *M. pelagios* possess a large, bulbous head and elongated body (Figure 1) whereas the head of *R. typus* is wide and dorsoventrally flattened, otherwise displaying similar body proportions to close relatives (Ebert, Dando, and Fowler 2021). *C. maximus* and *R. typus* have convergently evolved relatively lunate caudal fins and high aspect ratio pectoral fins associated with pelagic lifestyles (Sternes, Schmitz, and Higham 2024); whereas *M. pelagios* exhibits a more heterocercal caudal fin. Moreover, whilst *C. maximus* is a ram feeder, both *R. typus* and *M. pelagios* are engulfment feeders, producing low pressure in the buccal cavity to obtain small prey items (Martin 2007; Nakaya, Matsumoto, and Suda 2008; Sims 2000). Additionally, all three species also appear to have convergently evolved similar dental morphology (Mitchell 2016). Whilst the aforementioned biotic shifts in marine communities during the Paleogene provide viable (albeit speculative) adaptive explanations for the evolution of gigantism in filter‐feeding sharks, nothing is known about the mechanistic, developmental, and/or genetic basis of the various morphological and behavioral adaptations exhibited by filter‐feeding giants. Whilst *Aquilolamna milarcae*† also shares some of the morphological characteristics described above (Vullo et al. 2021), we herein focus on extant taxa due to a paucity of data regarding the species' ontogeny and feeding mechanism.

## 3 The Heterochrony Hypothesis

Proximate evolutionary explanations for adaptations form a fundamental component of our understanding of trait evolution (Thierry 2005). Here, we suggest that evolved changes in developmental timing (heterochrony) directly facilitated the evolution of morphological adaptations associated with filter‐feeding gigantism in lamniform sharks (Megachasmidae and Cetorhinidae). We focus on the unique craniofacial enlargement observed in both *C. maximus* and *M. pelagios* (Figure 1) that enables them to maximize prey capture and energetic intake, which is thought to be the limiting factor constraining body size in marine filter‐feeding vertebrates (Pimiento et al. 2019).

**Figure 2:**
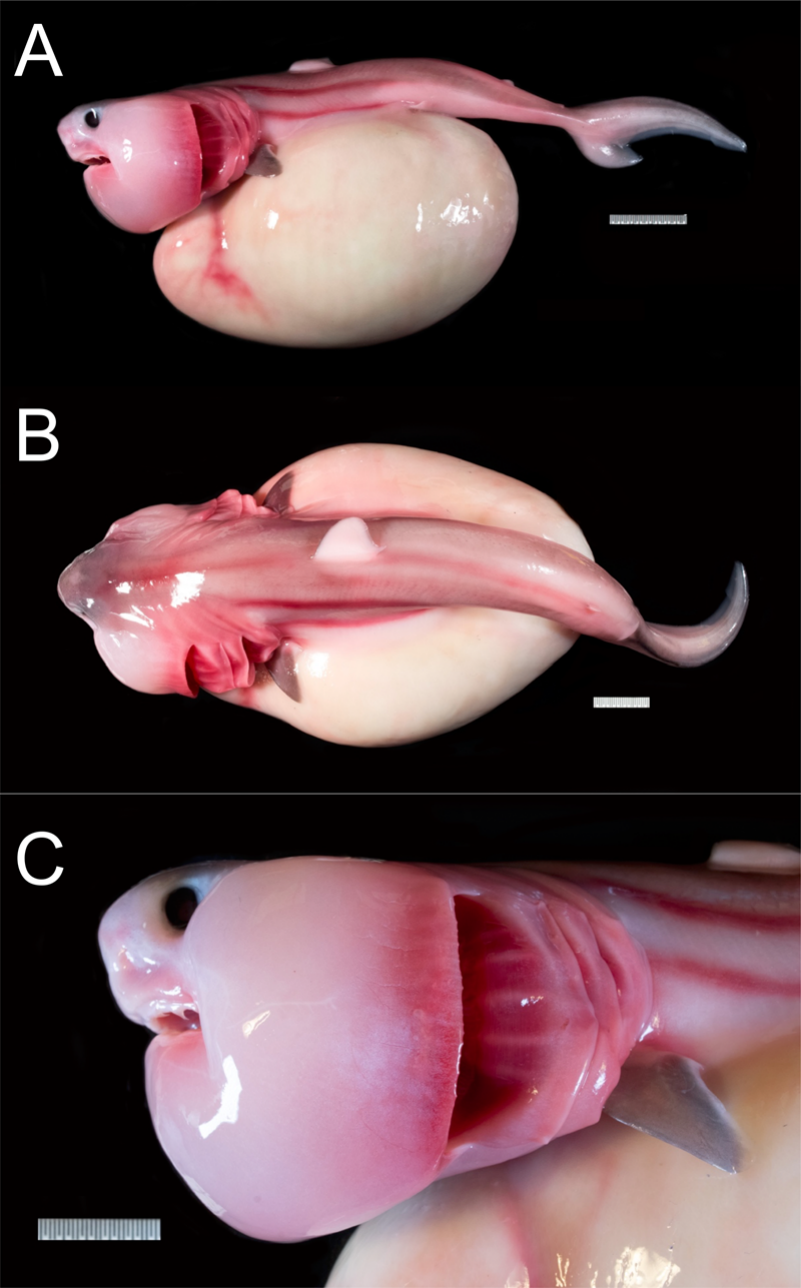
Porbeagle shark (*Lamna nasus*) embryo (specimen MCZ176738) in lateral (A, C) and dorsal (B) view. Images courtesy of Meaghan Sorce, Museum of Comparative Zoology. [Color figure can be viewed at wileyonlinelibrary.com]

As lamniform sharks, *C. maximus* and *M. pelagios* exhibit the aplacental viviparity mode of reproduction (Compagno 1990; Matthews 1950; Watanabe and Papastamatiou 2019). Alongside aplacental viviparity, lamniform taxa are known to be oophagous, a life history strategy in which unfertilized ova are ovulated and consumed by the first hatching embryos throughout gestation (Gilmore, Putz, and Dodrill 2011; Miller, Wails, and Sulikowski 2022). Oophagy imparts strong selection for certain morphological characteristics in lamniform embryos, most notably a greatly enlarged head with hypertrophied jaw adductor muscles (Figures 1 and 2) that closely resemble the head of adult filter feeders (*C. maximus* and *M. pelagios*), particularly in dorsal view (Figures 1 and 2B). Adults of both species also exhibit elongation of the body, more closely resembling the embryonic body form than other adult lamniform sharks such as *Lamna nasus*, that are comparatively stout‐bodied (Figures 1 and 2A). In embryos of oophagous species, craniofacial specialization maximizes foraging success by facilitating cannibalism and out‐competing of siblings in utero. We suggest, however, that these morphological characteristics, lost before parturition in non‐filter‐feeding lamniform sharks, are retained throughout ontogeny and co‐opted later in development to improve planktivorous foraging success in *C. maximus* and *M. pelagios*. Importantly, we are not suggesting that the specific craniofacial muscle architecture observed in embryonic forms (such as an enlarged quadratomandibularis) is necessarily conserved through ontogeny. Rather, retention of an enlarged, bulbous head (and potentially a proportionally elevated total muscle mass) and elongated trunk in adult forms may be accompanied by changes in the arrangement of jaw muscles consistent with a shift in functional demands from oophagy to filter feeding, without the clear and substantial shifts in head shape and body proportion through ontogeny seen in non‐filter feeding lamniform sharks.

**Figure 3:**
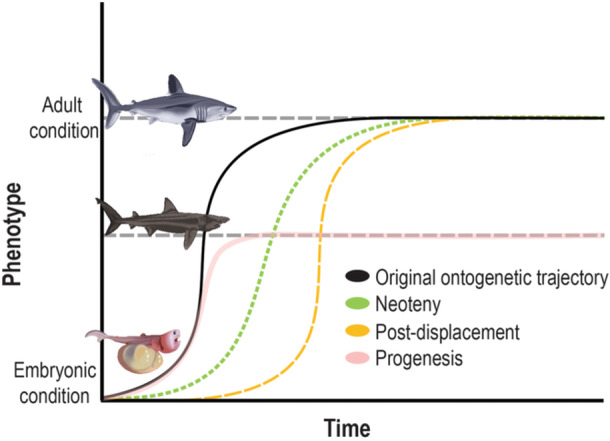
Original and paedomorphic ontogenetic trajectories reflecting changes in phenotype over developmental time. Decelerated development (neoteny) is denoted by the green dotted line; whereas, post displacement is denoted by the orange dashed line, and early sexual maturation (progenesis) is denoted by the pink faded line. Note that values for phenotype, time, and the magnitude of displacement and deceleration are arbitrary and for visualization purposes alone. Specific phenotypic start and end points and rates of development will vary across traits, species, and environmental contexts. [Color figure can be viewed at wileyonlinelibrary.com]

Retention of these embryonic characteristics in *C. maximus* and *M. pelagios* could have occurred by a process called heterochrony, a modification to the timeline of development (McNamara 2012). The enlarged bulbous head of lamniform embryos is clearly lost before birth in other lamniform taxa, such as mako (*Isurus oxyrhinchus*) and porbeagle (*Lamna nasus*) sharks, in favor of a more streamlined craniofacial morphology that is observed in all postnatal individuals and even in the latter stages of embryonic development (Ebert, Dando, and Fowler 2021; Joung and Hsu 2005; Tomita et al. 2018). Retention of embryonic or juvenile characteristics in the framework of heterochrony is referred to as paedomorphosis, which occurs through three principal mechanisms (Iordansky 2005; McNamara 1986): neoteny (a deceleration of development, i.e., a shallowing of the gradient of phenotypic change through time), post‐displacement (a delayed onset of development, with which subsequently proceeds as usual), and progenesis (early onset of sexual maturity and premature termination of phenotypic change, i.e., the original ‘adult’ phenotype is never achieved, despite development initially occurring as usual) (Figure 3). Theoretically, the construction of detailed ontogenetic growth curves would enable these mechanisms to be distinguished from one another (Figure 2); however, it is often a combination of neoteny, post‐displacement, and progenesis that results in the retention of juvenile characteristics (e.g., Denoël and Joly 2000; Tissot and Tissot 1988). The outcome of each of these mechanisms is ultimately that, at any given point in developmental time, a paedomorphic individual will exhibit a more ‘embryo‐like morphology’ than an individual following a standard ontogenetic trajectory (Figure 3).

Whilst data regarding ontogenetic growth curves in *C. maximus* and *M. pelagios* are limited, the available data are consistent with some combination of paedomorphic factors. For example, craniofacial enlargement similar to that observed in embryos of other lamniform sharks is present even in the smallest known individuals, and both *C. maximus* and *M. pelagios* show a gradual loss of this embryonic condition (i.e., a narrowing of the head) that continues well into adulthood (Ahnelt et al. 2020; Yun and Watanabe 2023). It is difficult (but not impossible, see “future studies” section) to determine which specific combination of neoteny, progenesis, and post‐displacement may have contributed to paedomorphosis in *C. maximus* and *M. pelagios*, as detailed, full‐body ontogenetic growth curves for these species (or other lamniform sharks such as *L. nasus*) are lacking. Our ability to discriminate between different ontogenetic trajectories is further complicated by uncertainty regarding whether sharks exhibit asymptotic or indeterminate growth (Heupel et al. 2014; Meekan et al. 2020). Whilst it cannot be ruled out conclusively, progenesis is unlikely given the continued narrowing of the head long after sexual maturity is reached (Ahnelt et al. 2020; Yun and Watanabe 2023) and because the age of *C. maximus* at sexual maturity ( ~ 6–8 years) does not seem to differ substantially from that of other large‐bodied lamniform sharks such as *Lamna ditropis* (3–10 years), *Carcharias taurus* (~10 years), *Alopias pelagicus* (6–9 years), and *I. oxyrinchus* (~4.5–8 years) (Frederickson, Cohen, and Berry 2016; Goldman and Musick 2006; Newbrey, 2013; Parker and Stott 1965).

Our heterochrony hypothesis thus suggests that neoteny or post‐displacement (or possibly progenesis) of embryonic craniofacial characteristics in *C. maximus* and *M. pelagios* enabled these sharks to retain a proportionally large mouth into adulthood, directly facilitating gigantism by ensuring that efficient prey acquisition could be maintained at a large body size. Without paedomorphosis, a proportionally narrow head (as exhibited by other lamniform sharks) may have rendered *C. maximus* and *M. pelagios* incapable of planktivorous gigantism, due to an inability to capture and process a sufficient volume of prey to satisfy their metabolic requirements. Whilst craniofacial enlargement is the most notable specialization of gigantic filter‐feeding lamniforms, these species also exhibit body elongation and, in the case of *M. pelagios*, a relatively heterocercal caudal fin. Both traits bear greater resemblance to the embryonic form relative to other lamniform species (Figures 1 and 2). Consequently, it is plausible that heterochrony may have contributed to the evolution of other aspects of morphology in these gigantic sharks, besides just the head.

If correct, the heterochrony hypothesis also explains the presence of the clear morphological differences between these two lamniform giants (*C. maximus* and *M. pelagios*) and *R. typus*, despite the broad ecological similarities between the three species (Ebert, Dando, and Fowler 2021). *R. typus* is an orectolobiform shark that does not exhibit oophagy (Pierce et al. 2021), and thus the rhincodontid lineage would not have exhibited the raw phenotypic variation necessary for filter‐feeding characteristics that are seen only to have evolved in lamniform sharks. It has been established that *R. typus* evolved both gigantism and its filter‐feeding habit independently of *C. maximus* and *M. pelagios* (Friedman et al. 2010; Pimiento et al. 2019), but our hypothesis also indicates that the evolution of this ecomorphotype could be underpinned by discrete developmental pathways in orectolobiform and lamniform sharks. Additional evo–devo studies (discussed later) will be needed to determine whether or not this is the case.

The heterochrony hypothesis is consistent with all existing morphological and developmental data for lamniform sharks, and indeed would not be the only case of craniofacial heterochrony driven by the evolution of novel trophic strategies. For example, comparing the ontogenetic growth trajectories for craniofacial characters across 27 species revealed that needlefish (Belonidae) jaws have undergone heterochronic shifts in morphology, likely due to shifts in the prey availability and consequently in the efficiency of planktivory (Boughton, Collette, and McCune 1991). In fact, heterochrony appears to underlie shifts in trophic morphology (including that of the jaws, gill rakers, and other aspects of the cranium) in a variety of marine fishes (Eastman et al. 2014; Gunter, Koppermann, and Meyer 2014; Hirt 2015; Kon and Yoshino 2002; Meyer 1987). These studies demonstrate that shifts in prey availability and trophic ecology can impart strong selective pressures on craniofacial morphology, triggering heterochrony which, in some cases, can result in jaw and head elongation. Indeed, other gigantic filter‐feeding taxa, namely, balaeonopterid whales, also exhibit paedomorphic cranial growth trajectories associated with enhanced prey acquisition (Lanzetti et al. 2023; Tsai and Fordyce 2014). This observation is particularly interesting given that non‐balaeonopterids exhibit contrasting growth trajectories (Lanzetti et al. 2023), and that Balaeonopteridae includes several of the largest known animals. Whilst the embryonic and adult phenotypes of balaeonopterid whales and lamniform sharks clearly differ, our heterochrony hypothesis would suggest that cranial paedomorphosis (that functions to maximize the efficiency of filter feeding) is a recurring theme among the largest chondrichthyan and cetacean lineages. Whilst these studies do not directly support the heterochrony hypothesis in the case of giant filter‐feeding lamniform sharks, they demonstrate that this mode of evolution is plausible and that paedomorphosis underlies morphological adaptations for filter‐feeding across the other two vertebrate clades to utilize this feeding strategy (Teleostii and Cetacea). In the latter case, it is plausible that this heterochrony may have played some role in the evolution of particularly large body sizes among balaeonopterid whales.

## 4 Future Studies and Testing the Hypothesis

Although our heterochrony hypothesis aligns with existing ontogenetic data for *M. pelagios* and *C. maximus* (Ahnelt et al. 2020; Yun and Watanabe 2023), future studies are needed to conclusively support or reject it. Crucially, our understanding of the gene regulatory networks that drive morphological variation in cartilaginous fishes remains limited (Gayford 2023; Gillis et al. 2022), impeding our ability to determine the mechanistic underpinnings of key morphological traits and the dynamics underlying fine‐scale trait evolution. In contrast, our understanding of the gene regulatory networks underlying morphological variation in bony fishes (Braasch et al. 2015; Peichel and Marques 2017), and indeed all other major vertebrate lineages (Böhmer, Rauhut, and Wörheide 2015; Boyko et al. 2010; Li et al. 2018; Mallarino and Abzhanov 2012; Vassilieva and Smirnov 2021) is comparatively more advanced. For such large (and rare, in the case of *M. pelagios*) species, direct evolutionary–developmental studies of putative heterochrony, such as those used to test heterochrony hypotheses in other clades (Gunter, Koppermann, and Meyer 2014) are currently out of reach. Even more traditional comparative approaches based on the slopes of ontogenetic trajectories (Boughton, Collette, and McCune 1991) require additional data, as detailed morphological descriptions of *C. maximus* and *M. pelagios* embryos and neonates are lacking from the literature. This impedes our understanding of how morphological characters change during early ontogeny in these species and the extent to which these changes differ from those seen in other lamniform species (Joung and Hsu 2005; Tomita et al. 2018).

However, there are several ways in which the heterochrony hypothesis could be tested, even without the further integration of genetic/genomic evo–devo approaches into chondrichthyan research. Arguably the most effective way to test the heterochrony hypothesis would be to develop rigorous, quantitative embryonic staging tables for *C. maximus*, *M. pelagios* (and other lamniform species), as has been done in other shark species (see Onimaru et al. 2018). Combined with measurement data from neonate, juvenile, and adult specimens, which could be sourced from fisheries, stranding events, or museum collections, this would allow ontogenetic growth trajectories comparable to those used to test for heterochrony in other clades to be constructed. This may also enable us to discriminate quantitatively between different mechanisms of heterochrony, such as neoteny and post‐displacement (Boughton, Collette, and McCune 1991). The increased usage of 3D imaging techniques such as computerized tomography or magnetic resonance imaging may also improve our understanding of fine‐scale anatomical transitions occurring through ontogeny in *C. maximus* and *M. pelagios*, and the extent to which they differ from those observed in other sharks.

A further question of interest regards whether the heterochrony hypothesis could apply to *A. milarcae*†. Similarly to *C. maximus* and *M. pelagios*, *A. milarcae*† exhibits a large, broad head and a comparatively narrow, elongated trunk (Vullo et al. 2021). However, this taxon is known only from isolated fossil remains, and many aspects of its ecology, growth, and anatomy remain ambiguous. Consequently, new fossil discoveries and paleontological analysis will be needed to address the extent to which the heterochrony hypothesis could apply to this enigmatic Cretaceous taxon.

In addition to testing the heterochrony hypothesis, more studies into the evolutionary causes of gigantism (i.e., both proximate and ultimate) in marine organisms are required. In the case of cartilaginous fishes, studies of body size evolution are restricted to Elasmobranchii, excluding Holocephali and stem chondrichthyans (Marion, Condamine, and Guinot 2024; Mull et al. 2024; Pimiento et al. 2019). Moreover, whilst filter feeding and heterothermy are thought to be the primary pathways toward gigantism, there are a number of shark lineages that exhibit large body size despite being ectothermic macropredators, such as *Galeocerdo*, *Somniosus*, and *Sphyrna* (Ebert, Dando, and Fowler 2021; Pimiento et al. 2019). These proposed relationships between body size and ecology are also not bidirectional, as many small‐bodied filter‐feeding elasmobranchs are known (Last et al. 2016). Expansion of existing comparative phylogenetic studies to consider additional potential drivers of gigantism and synergism between drivers may be sufficient to address this limitation. However, additional, proximate perspectives should also be considered, which will undoubtedly require greater integration of ontogenetic and developmental studies into the field of chondrichthyan evolutionary biology.

## 5 Conclusions

The giant filter‐feeding shark ecomorphotype has evolved on multiple occasions, apparently in association with periods of high primary productivity (Pimiento et al. 2019). Given the ecological and evolutionary significance of gigantism (Ferrón, Martínez‐Pérez, and Botella 2018; Vermeij 2016), developing an understanding of both the ultimate and proximate drivers of this ecomorphotype is important. The heterochrony hypothesis provides one such proximate explanation for the evolution of gigantism in filter‐feeding lamniform sharks. Whilst consistent with existing studies of ontogenetic allometry and the morphological differences between lamniform giants and *R. typus*, additional studies will be necessary to conclusively support or reject the heterochrony hypothesis. Proximate explanations merit consideration in studies of gigantism and body size evolution in Chondrichthyes, and the heterochrony hypothesis provides one example of how evolutionary changes to development could underpin morphological and morphometric trends observed in extant sharks and rays.

## Author Contributions

Joel H. Gayford conceived of the study. Joel H. Gayford and Jodie L. Rummer produced the figures. All authors contributed to the writing and reviewing of the manuscript.

## Conflicts of Interest

The authors declare no conflicts of interest.

## Data Availability

Data sharing is not applicable to this article as no data sets were generated or analyzed during the current study.
